# Lysosomal drug sequestration as a mechanism of drug resistance in vascular sarcoma cells marked by high CSF-1R expression

**DOI:** 10.1186/2045-824X-6-20

**Published:** 2014-10-01

**Authors:** Brandi H Gorden, Jhuma Saha, Ali Khammanivong, Gary K Schwartz, Erin B Dickerson

**Affiliations:** 1 Department of Veterinary Clinical Sciences, College of Veterinary Medicine, University of Minnesota, 1352 Boyd Avenue, Saint Paul, MN 55108, USA; 2 Laboratory of New Drug Development, Department of Medicine, Columbia University Medical Center, New York, NY, USA; 3 Herbert Irving Comprehensive Cancer Center, Columbia University Medical Center, New York, NY, USA; 4 Masonic Cancer Center, University of Minnesota, Minneapolis, MN, USA

**Keywords:** Angiosarcoma, Chemoresistance, CSF-1R, Endothelial, Hemangiosarcoma, Lysosome, Myeloid

## Abstract

**Background:**

Human angiosarcoma and canine hemangiosarcoma are thought to arise from vascular tissue or vascular forming cells based upon their histological appearance. However, recent evidence indicates a hematopoietic or angioblastic cell of origin for these tumors. In support of this idea, we previously identified an endothelial-myeloid progenitor cell population with high expression of endothelial cell markers and the myeloid cell marker, colony stimulating factor 1 receptor (CSF-1R). Here, we further characterized these cells to better understand how their cellular characteristics may impact current therapeutic applications.

**Methods:**

We performed cell enrichment studies from canine hemangiosarcoma and human angiosarcoma cell lines to generate cell populations with high or low CSF-1R expression. We then utilized flow cytometry, side population and cell viability assays, and fluorescence based approaches to elucidate drug resistance mechanisms and to determine the expression of hematopoietic and endothelial progenitor cell markers.

**Results:**

We demonstrated that cells with high CSF-1R expression enriched from hemangiosarcoma and angiosarcoma cell lines are more drug resistant than cells with little or no CSF-1R expression. We determined that the increased drug resistance may be due to increased ABC transporter expression in hemangiosarcoma and increased drug sequestration within cellular lysosomes in both hemangiosarcoma and angiosarcoma.

**Conclusions:**

We identified drug sequestration within cellular lysosomes as a shared drug resistance mechanism in human and canine vascular sarcomas marked by high CSF-1R expression. Taken together, our results demonstrate that studies in highly prevalent canine hemangiosarcoma may be especially relevant to understanding and addressing drug resistance mechanisms in both the canine and human forms of this disease.

## Introduction

Human angiosarcoma and canine hemangiosarcoma are aggressive malignancies of vascular tissue or vascular forming cells [1,2], and their morphology and pathological progression are virtually indistinct. Although doxorubicin remains the foundation of chemotherapy for humans and dogs, it provides minimal tumor control or survival benefit for angiosarcoma patients as only 16-36% of patients respond to treatment and over 50% of affected dogs still die within four to six months of their diagnosis [1,2]. In addition to their poor response rates, tumors in both species can be highly drug resistant. Thus, the outcomes of people and dogs are not likely to improve without an increased understanding of the biology and pathogenesis of these tumors. To complicate matters further, angiosarcomas occur infrequently restricting the study of their basic biology and clinical treatment [3]. However, the frequent occurrence of hemangiosarcomas in dogs (up to 7% of all canine malignancies) [4] provides a readily available resource to investigate tumor biology and explore related treatment options.

Identifying more successful treatment approaches may lie in addressing the cellular origin of these tumors rather than their histological appearance. Canine hemangiosarcoma and human angiosarcoma have been classified historically as tumors of malignant endothelium due to their histology and the expression of endothelial cell surface markers [2,5]. Based on the expression of early hematopoietic and endothelial progenitor markers, other studies have challenged this idea, suggesting instead that hemangiosarcomas might arise from bone marrow-derived angioblastic progenitors [6-8]. A similar analogy of progenitor cell origin has been drawn for human angiosarcoma [9].

In a more recent analysis, we identified and characterized a myeloid subpopulation from hemangiosarcoma cell lines that showed the expression of markers associated with bone marrow-derived myeloid progenitors (CD14 and CD115 or the colony stimulating factor 1 receptor, CSF-1R) co-expressed with surface markers associated with endothelial progenitor (CD34 and CD133) and endothelial cell differentiation (CD105, CD146, and α_v_β_3_) [8]. These cells also possessed phagocytic activity, and the co-expression of endothelial markers suggests a role in angiogenesis. Yoder *et al.* described a similar population of human myeloid cells that express a variety of hematopoietic (CD14, CSF-1R, and CD45) and endothelial markers (CD133, CD34, VEGFR2) and participate in blood vessel formation [10]. These cells possessed a myeloid progenitor cell activity and differentiated into phagocytic macrophages, but failed to contribute to the capillary endothelial layer *in vivo*. These similarities suggest that the myeloid-endothelial cell phenotype in hemangiosarcoma may represent a viable target for therapeutic intervention, and more specifically, targeting of CSF-1R.

CSF-1R and its ligands, CSF-1 and IL-34, are commonly associated with the survival, proliferation, differentiation, and activation of mononuclear phagocytes [11-15]. CSF-1R expressed by tumor associated macrophages (TAMs) can have therapeutic implications since TAMs impact tumor growth by promoting myeloid cell-mediated angiogenesis, chemoresistance, and metastatic spread [16-20]. However, expression of CSF-1R by tumor cells also indicates a non-macrophage functional role for the receptor. In this regard, Cioce *et al.* reported increased expression of CSF-1R mRNA in mesothelioma versus normal tissue specimens and demonstrated that CSF-1R expression identified chemoresistant cells in both primary cultures and mesothelioma cell lines [21]. Thus, CSF-1R expression may serve as a marker to identify drug resistant populations in some cancers.

For this study, we demonstrate that both hemangiosarcoma and angiosarcoma cells with high expression of CSF-1R are more drug resistant than their CSF-1R low-expressing counterparts, indicating a shared mechanism for the observed treatment failures and subsequent drug resistance. Our data also suggest that part of this resistance may be achieved through drug sequestration within cellular lysosomes. Intriguingly, drug resistance in canine hemangiosarcoma is associated with CD133 expression, suggesting that resistance may be associated with a stem or progenitor cell phenotype and may be related to the degree of cellular differentiation. Further characterization of these cells and utilization of approaches to disrupt lysosomal drug trapping could improve drug responses as well as treatment outcomes.

## Materials and methods

### Cell culture

The DD-1 cell line was derived from a splenic hemangiosarcoma [22], and the COSB line was derived from a xenograft of the original cell line, SB-HSA [23]. The AS5 human angiosarcoma cell line was derived from a primary angiosarcoma of the thigh [24]. All cell lines were cultured as described previously [6,22,25]. Cells were maintained in culture for up to 8 weeks before new vials were thawed to ensure similar passage numbers were used for all experiments.

### Flow cytometry and magnetic enrichment

The primary antibodies used were: anti-CSF-1R (CD115)-Cy5.5 (Bioss Inc., Woburn, MA), anti-CD34-Alexa Fluor 647, anti-CSF-1R-RPE (AbD Serotec, Raleigh, NC); anti-CD117(c-kit)-PE and APC, anti-CD34-PE, anti-CD243(ABCB1)-PE and APC, anti-CD338 (ABCG2)-PE and APC (eBioscience, San Diego, CA), anti-CD34-APC (human) (eBioscience), anti-CD34-PE and APC (canine) (eBioscience), and anti-CD133/AC133-PE and APC (Miltenyi Biotech, Auburn, CA). The detection of cell surface markers was carried out as described, previously [8]. Data were collected on a BD FACS Calibur or a BD Accuri C6 flow cytometer (BD Biosciences, San Jose, CA) and then analyzed using FlowJo software. To exclude dead cells from analysis, 7-AAD was added to cells 10 minutes before acquisition, and compensation was performed to account for 7-AAD fluorescence in FL2. CSF-1R^low^ and CSF-1R^high^ populations were enriched from hemangiosarcoma and angiosarcoma cells by staining 2 × 10^7^ to 4 × 10^7^ cells with anti-CD115-RPE antibody followed by magnetic separation using the EasySep PE-Selection Kit (Stemcell Technologies, British Columbia, Canada) according to the manufacturer’s instructions. Cells were analyzed immediately after enrichment by flow cytometry to determine the percent enrichment from the original cell line culture.

### Phagocytosis assay

Cells were plated in triplicate using 10,000 cells per well in 100 μL of culture medium. FITC-conjugated, rabbit-IgG-coated latex beads (Cayman Chemical Company, Ann Arbor, MI) were added to the cells. No beads were added to negative control wells. The emission at 535 nm was measured for each well after 24 hours using a Wallac Victor2 1420 Multilabel Counter. Relative phagocytosis for each cell line was determined by dividing the fluorescence of the wells with beads by the fluorescence of the respective negative controls.

### Cytotoxicity assays

Cells were plated in triplicate at 2,500 (DD-1), 5,000 (COSB) or 10,000 (AS5) cells per well in 100 μL of culture medium and exposed to increasing concentrations of doxorubicin (Bedford Laboratories, Bedford, OH). Cell viability was determined 72 hours later using the colorimetric Cell Titer 96® Aqueous Non-Radioactive Cell Proliferation Assay (MTS Assay; Promega, Madison, WI) according to the manufacturer’s instructions. In order to compare the percent viability between the CSF-1R^low^ and the CSF-1R^high^ populations and reduce artifacts due to potential differences in metabolic properties between cell populations, standard curves were generated for each CSF-1R population in each cell line, and the cell viability was determined based upon the total cell number from the standard curve. A separate standard curve for the CSF-1R^low^ and the CSF-1R^high^ populations was generated for each experiment.

### Side population analysis

Filtered DD-1 or COSB cells were incubated in the presence or absence of 10 μM verapamil for 15 minutes at 37°C. DyeCycle Violet (DCV) (Life Technologies, Eugene, OR) was added to a final concentration of 10 μM, and the cells were incubated for an additional 60 minutes at 37°C with intermittent mixing. Cells were washed, filtered, and maintained on ice until analysis. Propidium iodide was added to each sample immediately before collection to exclude dead cells from analysis. DCV emission was detected using a BD LSRII flow cytometer (BD Biosciences). Verapamil was used to determine the SP gates, and data were analyzed using FlowJo software (Tree Star Inc.).

### Doxorubicin assays and detection

Cells were plated at 100,000 cells per well in 3 mL of culture medium in a 6-well plate. After 2 hours, cells were treated with 1 μM doxorubicin for 1 hour, washed with PBS, and stained for CSF-1R expression using a Cy5.5 labeled CSF-1R antibody at t = 0 and 24 hours. Intracellular doxorubicin was measured according to established methods [26,27]. To exclude dead cells from analysis, 7-AAD was added to filtered cells 10 minutes before data acquisition and compensation was used to account for the fluorescence of 7-AAD in FL2.

To determine the percent viable cells positive for CSF-1R expression after doxorubicin exposure, COSB cells were plated in triplicate as described for the cytotoxicity assays. Cells were exposed to increasing concentrations of doxorubicin (Bedford Laboratories) for 72 hours and then harvested. Cells were stained for CSF-1R expression using a Cy5.5 labeled CSF-1R antibody and 7-AAD was added to the cells before analysis.

### LysoTracker detection

Cell lines were grown until they were approximately 80% confluent and then harvested for analysis. The cells were blocked with normal rat serum in cold PBS containing 2% fetal bovine serum (Atlanta Biologicals, Atlanta, GA) and 2 mmol/L EDTA (Sigma, St. Louis, MO) for 2 minutes, and then incubated with an anti-CSF-1R-RPE antibody (AbD Serotec) for 30 minutes on ice. After incubation, the cells were washed with cold PBS to remove serum and incubated with increasing concentrations of LysoTracker® Deep Red (Life Technologies) in serum free medium on ice for 10 minutes. The cells were centrifuged and washed with cold PBS. To exclude dead cells from analysis, 7-AAD or LIVE/DEAD® Cell Stain (Life Technologies) was added to the cells before acquisition on an Accuri or BD LSRII flow cytometer (BD Biosciences). The CSF-1R^high^ population (~1-2% of the total cell population) was identified using anti-CSF-1R RPE labeling. Approximately 3-5% of the dimmest cell population was used to represent the CSF-1R^low^ population. The CSF-1R populations were normalized for comparison by subtracting the background (cells without LysoTracker, negative control) and then dividing the median fluorescence intensity of each concentration of LysoTracker by the median fluorescence intensity of the each cell population without LysoTracker (negative control). Values are presented as relative fluorescence levels for comparison.

### Statistical analysis and data presentation

All EC_50_ calculations were made using Prism 5 Software (GraphPad Inc. San Diego, CA) using a 4-parameter curve fit. Bar graphs are presented as blank-subtracted (adjusted) means ± SD. Comparisons between groups were made using a two-sided Student’s *t-*test to evaluate statistical significance, and a p-value ≤ 0.05 was considered significant.

## Results

### CSF-1R^high^ populations can be enriched from hemangiosarcoma and angiosarcoma cell lines and hemangiosarcoma CSF-1R^high^ cells display increased phagocytosis

**Figure 1:**
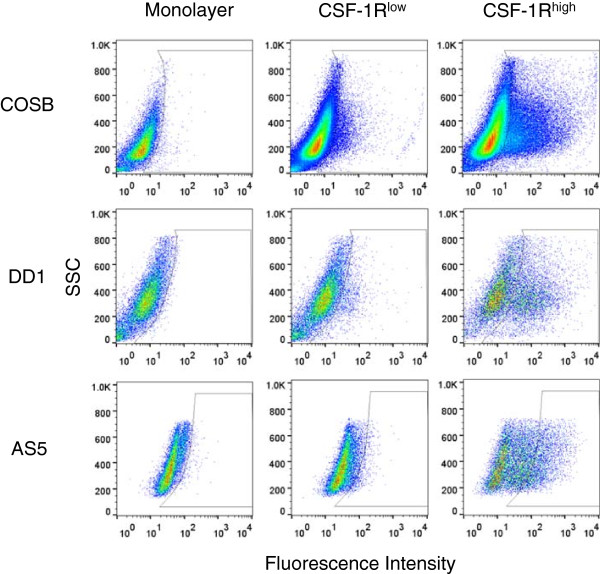
**Enrichment of cells expressing CSF-1R (CSF-1R**^**high**^**) from canine hemangiosarcoma and human angiosarcoma cell lines.** Monolayer cells were labeled with an RPE-CSF-1R antibody and enriched for CSF-1R expression using magnetic bead separation. Cell populations were assessed by flow cytometry to determine the percentages of cells in each population showing low (CSF-1R^low^) or high (CSF-1R^high^) expression of the receptor.

Cell surface expression levels of CSF-1R were determined for the canine hemangiosarcoma cell lines COSB and DD-1 and the human angiosarcoma cell line AS5 by flow cytometry. Flow cytometry analysis showed that a small percentage of cells (typically 1-2% of the cell monolayer) expressed high levels of CSF-1R in each cell line whereas the majority of the cells showed little to no expression (Figure 1). To obtain a greater number of cells expressing high levels of CSF-1R (CSF-1R^high^) for phenotypic and functional assays, we used CSF-1R antibody staining and enrichment with magnetic beads to select for cells with high CSF-1R expression as well as to obtain a subset of cells with low expression (CSF-1R^low^) (Figure 1). The percentage of CSF-1R^high^ cells in the enriched populations ranged from ~20-40% depending on the cell line. As an example, a summary of the enriched populations presented in Figure 1 is presented in Additional file 1: Table S1.

**Figure 2:**
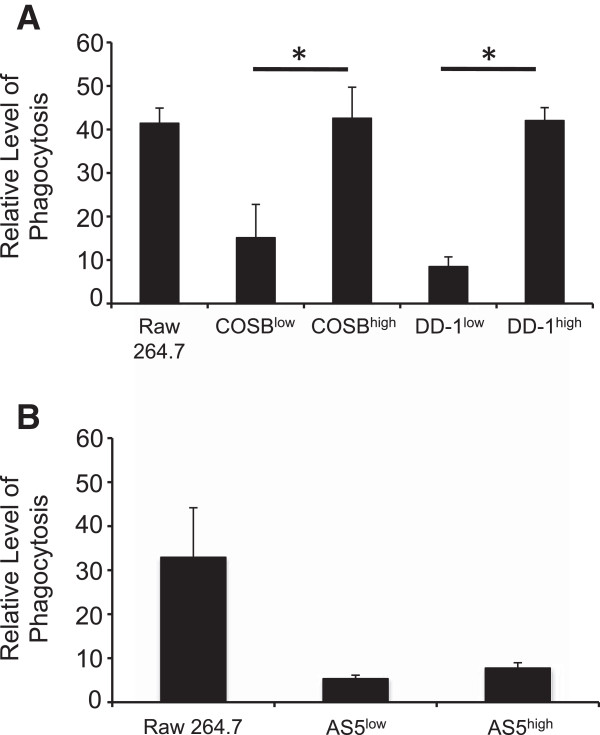
**CSF-1R**^**high **^**cells exhibit increased phagocytosis.** Phagocytosis of FITC-conjugated, IgG-coated latex beads was assessed in **(A)** CSF-1R^low^ and CSF-1R^high^ cells in hemangiosarcoma cell lines and **(B)** in the angiosarcoma cell line, AS5. Phagocytosis is represented as relative fluorescence intensity by each cell line. The macrophage cell line, RAW264.7, was used a positive control for the assay. The increased phagocytic activity in the CSF-1R^high^ populations from the hemangiosarcoma cell lines was significant (COSB, p* = 0.003 DD-1, p* = 0.034) compared to the activity detected in CSF-1R^low^ populations. Statistical significance was not detected (p = 0.08) between the CSF-1R^low^ and the CSF-1R^high^ cell populations enriched form the AS5 cell line.

To determine if the CSF-1R^high^ cells had retained or acquired myeloid cell properties, we determined the capacity of CSF-1R^low^ and CSF-1R^high^ cells for phagocytosis. Significant increases in phagocytic activity in the CSF-1R^high^ cells enriched from the COSB (p = 0.003) and the DD-1 (p = 0.034) cell lines were observed when compared to their CSF-1R^low^ counterparts, reaching a level comparable to that observed in the mouse macrophage cell line RAW 264.7 (Figure 2A). Phagocytosis also was evident for the CSF-1R^low^ and CSF-1R^high^ populations from the AS5 cell line. Although the levels of phagocytosis were similar to those observed for the DD-1 CSF-1R^low^ cell population, the difference between the CSF-1R^high^ and the CSF-1R^low^ cell populations was not significant (p = 0.08) (Figure 2B). These results demonstrate that enriched CSF-1R^high^ populations from hemangiosarcoma and angiosarcoma cell lines can be obtained, and that CSF-1R^high^ cell populations from the hemangiosarcoma cell lines retain a myeloid-like character. Phagocytosis by endothelial cells also has been described [28], and this is in keeping with the myeloid-endothelial phenotype previously identified for hemangiosarcoma cell lines [6,8].

### High CSF-1R expression correlates with increased resistance to doxorubicin

**Figure 3:**
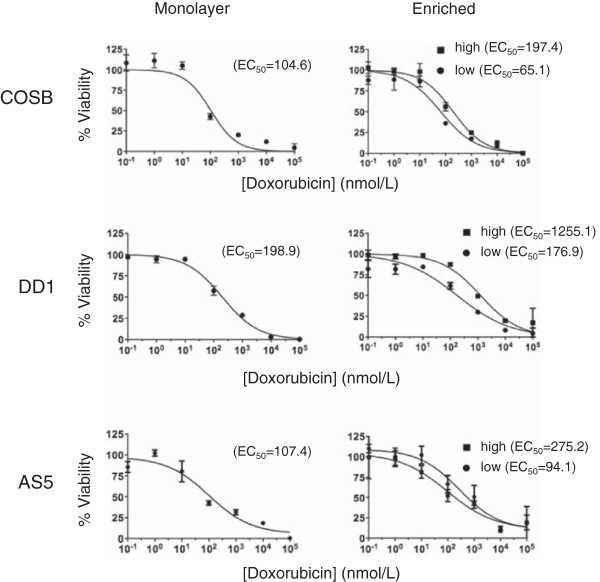
**CSF-1R**^**high **^**cells show increased resistance to standard chemotherapy agents.** Unenriched monolayer cells, CSF-1R^low^, or CSF-1R^high^ cells enriched from hemangiosarcoma or angiosarcoma cell lines were treated with increasing concentrations of doxorubicin for 72 hours. The relative viability was assessed using an MTS assay. The approximate EC_50_ values for each subpopulation are shown in parenthesis from representative experiments. All conditions were performed in triplicate and error bars represent standard deviation.

To determine if CSF-1R^high^ cells display differential drug sensitivities versus the monolayer cell and CSF-1R^low^ cell populations, we conducted comparative assays using doxorubicin, which is used commonly to treat both canine hemangiosarcoma and human angiosarcoma [1,2]. The relative viabilities of the monolayer, CSF-1R^low^, and CSF-1R^high^ cell populations in the presence of increasing doxorubicin concentrations are shown in Figure 3. In each case, the CSF-1R^high^ cell populations were more resistant to doxorubicin when compared to both the monolayer and CSF-1R^low^ cell populations. The approximate EC_50_ values for each population are shown in parentheses. The EC_50_ values for the monolayer cell populations were similar to, yet always slightly higher than, those determined for the CSF-1R^low^ cells. This result is consistent with the idea that the CSF-1R^low^ cells make up the bulk of the monolayer cell culture, but that the contribution of the CSF-1R^high^ cells to drug resistance can be observed. Non-overlapping 95% confidence interval values for the CSF-1R^low^ and CSF-1R^high^ cell populations enriched from the COSB and DD-1 cell lines (Additional file 1: Table S2) provide support for the observed drug sensitivities. While overlap of the 95% confidence intervals for the CSF-1R^low^ and CSF-1R^high^ cell populations from the AS5 cell line was noted, the differences in drug sensitivities were consistently reproducible in our assays.

To further verify that the CSF-1R^high^ cells are responsible for the observed increase in drug resistance, we treated the COSB cell line with the increasing concentrations of doxorubicin for 72 hours and determined the CSF-1R-expression by the cells remaining after treatment (Additional file 2: Figure S1A and B). At the lowest concentrations of doxorubicin, CSF-1R^low^ cells comprised the majority of the cell population, but the number of CSF-1R^low^ cells diminished as the concentration of doxorubicin increased. At the highest concentrations, the cell populations were comprised predominately of cells expressing CSF-1R, indicating that CSF-1R expression can be utilized as a marker for drug resistant cell populations.

### CSF-1R^high^ cells from hemangiosarcomas express high levels of ABCB1 and ABCG2 but show limited dye efflux from their side populations

ABC transporters can efflux chemotherapy agents from cancer cells to reduce intracellular drug accumulation and prevent high level of drugs from reaching their intended intracellular targets. Because doxorubicin can be effluxed by the transporters ABCB1 (P-glycoprotein or P-gp) or ABCG2 (breast cancer resistance protein or BCRP) [29,30], we chose to examine the expression of ABCB1 and ABCG2 in our cell lines and determine if drug efflux might contribute to the observed resistance in the CSF-1R^high^ populations.

**Figure 4:**
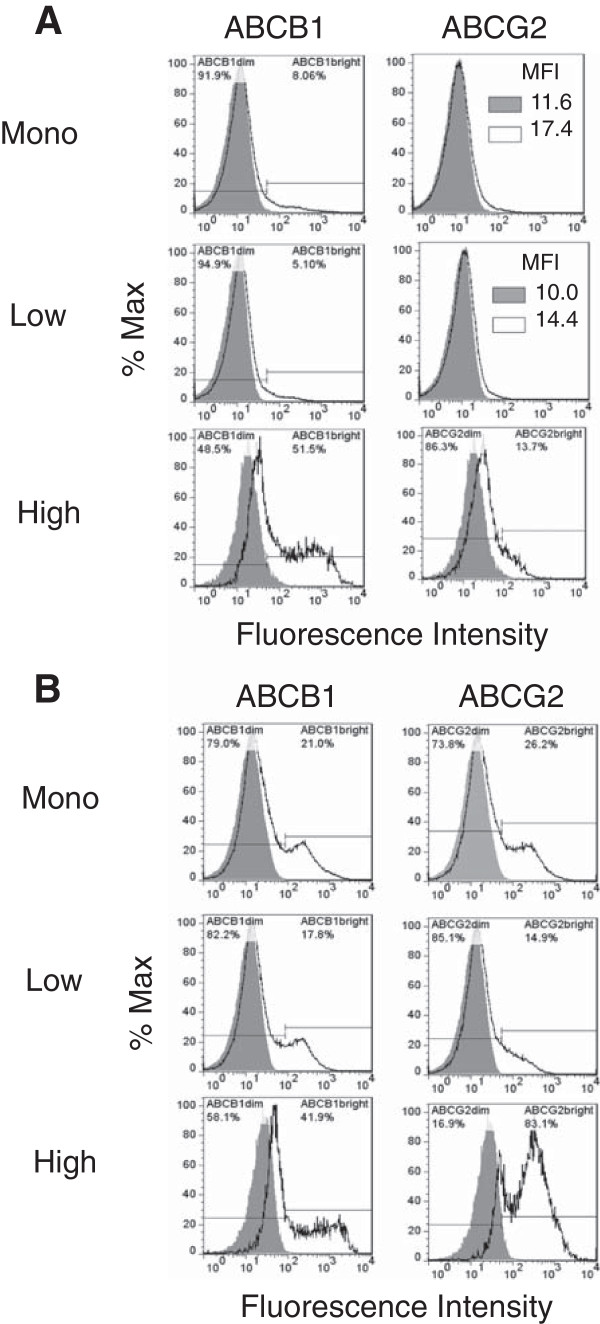
**Canine hemangiosarcoma cells express ABCB1 and ABCG2. (A)** Cell surface expression of the ABC transporters by COSB and **(B)** DD-1 cells was assessed using flow cytometry. Positive staining is indicated by the solid black lines, and the isotype controls are represented as shaded regions. MFI = mean fluorescence intensity.

We first examined ABC transporter expression in the canine hemangiosarcoma cell lines (Figure 4A and 4B). The expression levels of ABCB1 and ABCG2 in the monolayer and the CSF-1R^low^ cell populations from the COSB cell line were relatively low (Figure 4A). However, high expression of ABCB1 was displayed by a small percentage of the cells (approximately 5-8% of the total cell population), and these cells were designated as "bright" on the histograms to distinguish highly positive cells from those with low ("dim") transporter expression. ABCB1 and ABCG2 expression was detected in the monolayer and the CSF-1R^low^ cell populations from the DD-1 cell line, and the expression patterns between the monolayer and CSF-1R^low^ cells were similar (Figure 4B). The COSB and DD-1 CSF-1R^high^ cell populations showed increased expression of ABCB1 and ABCG2 overall, and both populations displayed a bimodal expression pattern with substantial increases in the percentage of "bright" cells. In contrast, ABCB1 and ABCG2 expression was low or not detected in the populations from the AS5 cell line, and the expression was restricted to the monolayer and CSF-1R^low^ cell populations (Additional file 2: Figure S2). Our results suggest that ABCB1 and ABCG2 expression might contribute to the higher doxorubicin resistance observed for the hemangiosarcoma CSF-1R^high^ cell populations; however, other transporters or mechanisms appear to be involved in drug resistance in the AS5 cell line.

**Figure 5:**
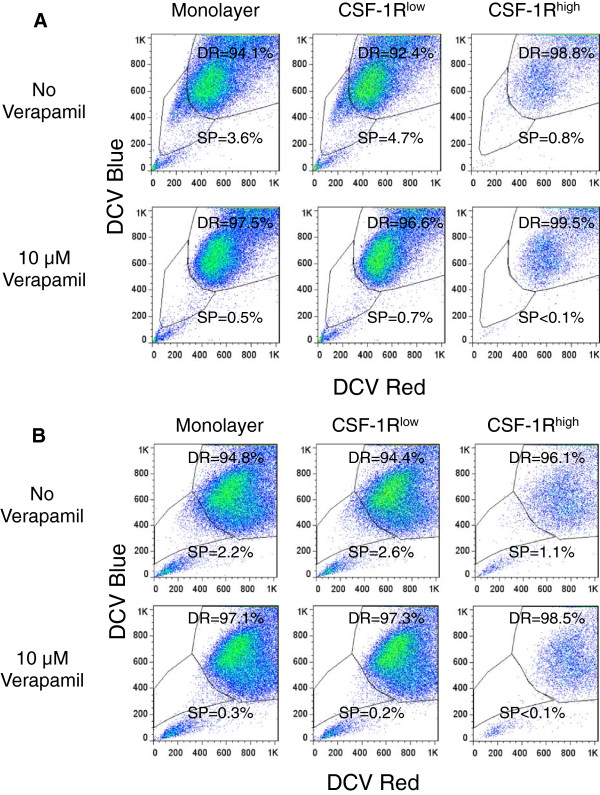
**Dye exclusion identifies side populations in unenriched monolayers, CSF-1R**^**low**^**, and CSF-1R**^**high **^**populations. A**. DyeCycle Violet efflux was measured in monolayer, CSF-1R^low^, and CSF-1R^high^ cells to identify side population (SP) and dye retaining (DR) cells by COSB and **(B)** DD-1 cells. Verapamil was used to inhibit dye efflux. Propidium iodide was added immediately before analysis of all samples in order to exclude dead cells from analysis.

To further clarify the potential roles of ABCB1 and ABCG2 in the CSF-1R^high^ cell-associated drug resistance, we performed side population analysis using DyeCycle Violet (DCV). DCV is a viable dye known to be effluxed from cells by ABCB1 and ABCG2 [31,32]. We did not perform this assay using the AS5 cells due to the low cell surface expression of the ABC transporters. Both the monolayer and CSF-1R^low^ cell populations from the COSB and DD-1 cell lines contained dye effluxing populations ranging from approximately 2.2% (DD-1, monolayer cells) up to about 4.7% (COSB CSF-1R^low^) of the total cells (Figure 5A and B). These side populations were highly sensitive to verapamil, which is a broad inhibitor of ABC transporter activity that primarily affects ABCB1 activity but affects ABCG2 activity at high concentrations [30,32].

Although the side populations were almost completely eliminated by verapamil in all cases, the dye excluding populations identified in the CSF-1R^high^ cells (~1%) were smaller than those observed in the monolayer and the CSF-1R^low^ cells (~2-5%). This was unexpected given the more abundant expression of ABCB1 and ABCG2 by the CSF-1R^high^ populations. This result prompted us to look for other mechanisms of drug resistance since the level of dye efflux (and potentially the level of ABC transporter expression) by these cells may not correspond to drug sensitivity.

### CSF-1R^high^ cells show higher levels of intracellular doxorubicin and LysoTracker suggesting increased lysosomal drug sequestration

**Figure 6:**
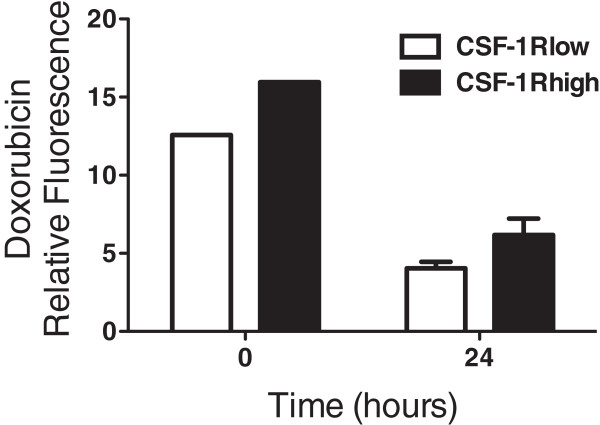
**CSF-1R**^**high **^**cells show increased retention of doxorubicin.** COSB cells were incubated with 1 μM doxorubicin and the levels of doxorubicin and CSF-1R expression were measured by flow cytometry immediately after incubation (t = 0) and 24 hours later (t = 24). The levels of doxorubicin were significantly higher in the CSF-1R^high^ cells than in the CSF-1R^low^ cells at t = 0 (p = 0.005) and t = 24 hours (p = 0.03).

Doxorubicin has been shown to accumulate in cellular lysosomes in a variety of cell types, including those of myeloid lineage [33,34]. Based on the drug resistant and myeloid-like character of the CSF-1R^high^ cells, we compared intracellular doxorubicin levels in CSF-1R^low^ and CSF-1R^high^ cells from the COSB cell line. Cells were incubated with doxorubicin for 1 hour followed by washing and further incubation for 24 hours in drug-free medium. The relative levels of doxorubicin remaining in both subpopulations were analyzed by flow cytometry to detect doxorubicin fluorescence. The levels of doxorubicin were significantly higher in the CSF-1R^high^ cells than in the CSF-1R^low^ cells at t = 0 (p = 0.005) and t = 24 hours (p = 0.03) (Figure 6).

**Figure 7:**
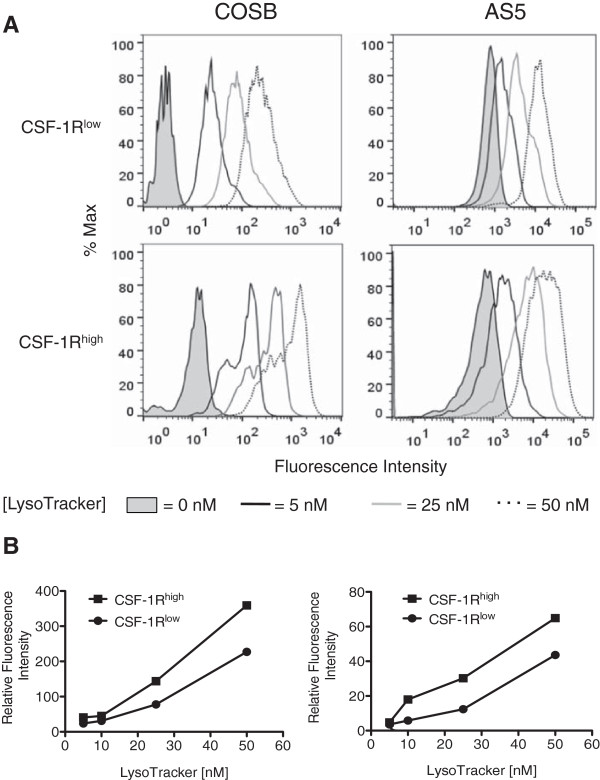
**CSF-1R**^**high **^**cells preferentially accumulate higher levels of LysoTracker. (A)** COSB and AS5 were stained for cell surface CSF-1R expression using an RPE-labeled CSF-1R antibody followed by incubation with increasing concentrations of LysoTracker DeepRed. LysoTracker fluorescence was assessed in the CSF-1R^high^ cell population (approximately 1-2% of all cells), and this fluorescence was compared to the CSF-1R^low^ cell population (dimmest 3-5% of all cells) in each cell line. The fluorescence intensity of cells without LysoTracker or with increasing concentrations of LysoTracker added to the CSF-1R^low^ and CSF-1R^high^ cell populations is shown. For the AS5 studies, the coefficient of variation (CV) values are provided as [LysoTracker concentration (CSF-1R^low^, CSF-1R^high^)] for comparison of the histograms: [0 nm (39.4, 88.9); 5 nm (61.2, 109); 25 nM (78.3, 97.6); 50 nM (65.2, 86.3] **(B)** The relative fluorescence levels for each population compared to the untreated controls were plotted against the increasing LysoTracker concentrations. The graphs represent the data presented from each cell line in panel **A**.

Based on these data, we surmised that the CSF-1R^high^ cells might have a higher lysosomal capacity for drug sequestration than their CSF-1R^low^ counterparts. To test this, we labeled the CSF-1R^high^ cells in the COSB and AS5 cell lines with the same antibody used for the cell enrichment process. This was followed by incubation with increasing concentrations of LysoTracker DeepRed in order to compare the relative lysosomal sequestration capacity between the cell subpopulations. Both the CSF-1R^low^ and the CSF-1R^high^ cell populations showed an increase in relative fluorescence as the LysoTracker concentration was increased; however, the relative LysoTracker fluorescence in the CSF-1R^high^ cells ranged from approximately 1.5- to 2.5-fold higher between all concentrations tested (Figure 7A and B and Additional file 1: Table S3). Our data also indicate that the CSF-1R^high^ cell population is more heterogeneous than the CSF-1R^low^ population. This is evident in the COSB CSF-1R^high^ cell population (Figure 7A) where a bimodal distribution becomes pronounced as the concentration of LysoTracker is increased; however a small "high" population also is evident in the CSF-1R^low^ cells. For the AS5 cell line, the histograms were broader for the CSF-1R^high^ populations compared to those from the CSF-1R^low^ populations, potentially indicating a wider distribution of lysosomal capacity and heterogeneity.

Our results show that cells with high CSF-1R expression enriched from hemangiosarcoma and angiosarcoma cell lines are more drug resistant than cells with low expression of the receptor. The data suggest that this resistance may be achieved, in part, through increased ABC transporter expression and/or drug sequestration within cellular lysosomes. Our results also suggest the idea that the CSF-1R^high^ cells have a greater lysosomal capacity, and further isolation via cell sorting and characterization these cells might be achieved using dual staining of CSF-1R^high^ in combination with LysoTracker analysis.

### CSF-1R^high^ cells show exclusive expression of CD133

**Figure 8:**
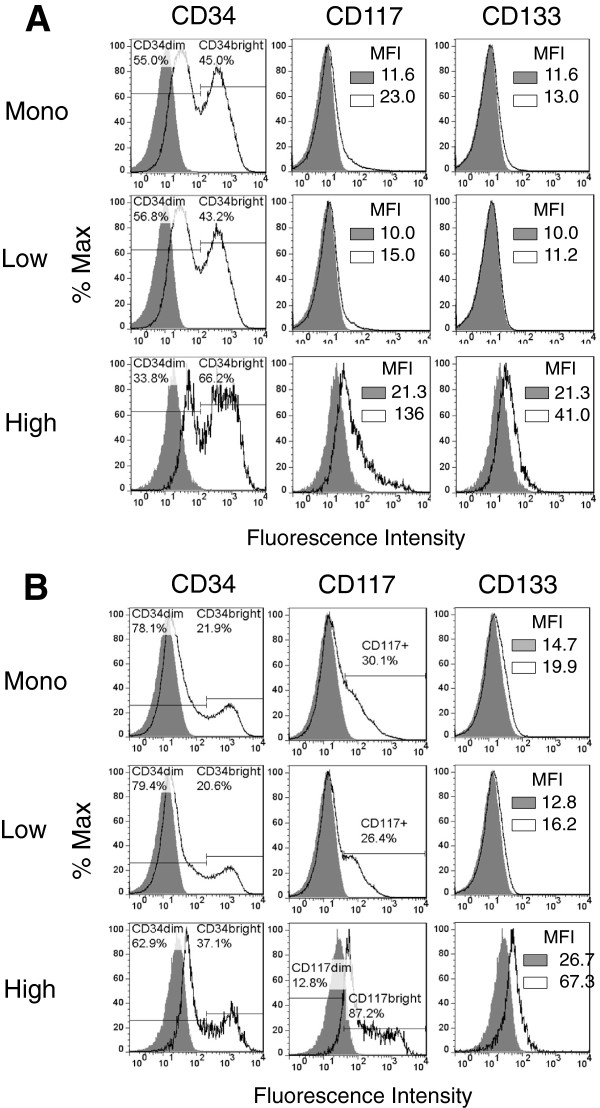
**Canine hemangiosarcoma cells express CD34, CD117, and CD133. (A)** Cell surface expression of CD34, CD117, and CD133 by COSB and **(B)** DD-1 cells was assessed using flow cytometry. Positive staining is indicated by the solid black lines, and the isotype controls are represented as shaded regions. MFI = mean fluorescence intensity.

Finally, we sought to determine if CSF-1R was co-expressed with the stem and progenitor cell markers CD34, CD117, and CD133, since these markers have been associated with tumor initiating cells and as well as drug resistance [35,36]. A bimodal expression pattern for CD34 was noted in all of the canine hemangiosarcoma cell populations (Figure 8A and B). In particular, CSF-1R^high^ cells had a higher percentage of CD34^bright^ cells than cells from the unenriched monolayers and CSF-1R^low^ subsets. Expression of CD117 was variable in the CSF-1R^low^ populations from COSB and DD-1, but higher expression was noted in the CSF-1R^high^ populations. A bimodal expression pattern for CD117 also was observed in the DD-1 cell line, indicating heterogeneity within the CSF-1R^high^ cell population. Interestingly, CD133 expression was restricted to the CSF-1R^high^ cell population in both canine cell lines, indicating the CD133 expression also may be used to distinguish drug resistant cells. In contrast, expression of CD133 was not observed in the AS5 angiosarcoma cell line (Additional file 2: Figure S3). However, CD34 and CD117 were expressed in all of the cell populations although higher expression was observed in the monolayer and CSF-1R^low^ cell populations.

## Discussion

Human angiosarcoma and canine hemangiosarcoma are aggressive vascular tumors where there are currently no effective treatments [2,3,37]. Angiogenesis and inflammation are key features of hemangiosarcoma [7,8], and progenitor cell populations expressing markers of both endothelial and myeloid progenitors support this premise [8]. Because these cells may participate in both the angiogenic and inflammatory responses, a better understanding of the cell biology would provide insight into new therapeutic opportunities. Here, we show that cell populations enriched for the myeloid marker, CSF-1R, are highly drug resistant in hemangiosarcoma and angiosarcoma, and this resistance may be mediated through drug sequestration within cellular lysosomes.

Lysosomes have been shown to be involved in the sequestration of amine-containing drugs such as doxorubicin [38,39]. The sequestration of drugs away from cellular target sites into cytoplasmic organelles prevents the drugs from reaching their targets of interest, leading to ineffective drug therapy. In support of this mechanism, our results suggest that a small population of cells identified by CSF-1R expression is more resistant due to doxorubicin sequestration into what are likely cellular lysosomes. While further studies are needed, the more myeloid-like character of the CSF-1R^high^ cells may play a role. Prior studies using human myeloid leukemia cell lines demonstrated that the more drug resistant variants of the lines showed increased drug sequestration within lysosomes [33,34]. As a consequence of the increased lysosomal drug trapping, nuclear accumulation of the drug was decreased leading to lower cytotoxicity [34].

More recently, Sukhai *et al.* showed that lysosomes isolated from primary human acute myeloid leukemia (AML) cells, CD34^+^ AML progenitor cells, as well as human and mouse AML cell lines contained larger lysosomes when compared to lysosomes found in normal human CD34^+^ hematopoietic cells [40]. Although larger in size, the number of lysosomes per cell did not differ significantly between AML cells and normal cells. Alterations in lysosomal size may be indicative of altered metabolic processes, specifically alterations in fatty acid metabolism [41], which may be important for altered energy needs of cancer cells. Furthermore, primary human AML and AML progenitor cells were more sensitive to the antimalarial agent mefloquine, a quinoline approved for the treatment of malaria and known to accumulate in the lysosomes of the malarial parasite [42]. These observations provide the rationale for therapeutically targeting the lysosomal compartment in AML, and they also support the investigation of the effects of lysosomal disruption on hemangiosarcomas and angiosarcomas since this approach may target the more drug resistant CSF-1R^high^ cells.

While it is likely that doxorubicin is sequestered in cellular lysosomes largely due to its lysosomotropic properties [34], drug sequestration within lysosomes also may be due to ABC transporter activity. Chapuy *et al.* determined that the ABCA3 transporter expression was localized to lysosome and multivesicular body membranes in AML, and that ABCA3 expression was associated with unfavorable treatment outcomes for AML patients [43]. Further analysis in chronic myeloid leukemia (CML) showed that lysosomal storage capacity was increased with increases in ABCA3 expression, indicating that ABCA3 may contribute to drug resistance by facilitating lysosomal drug sequestration [44]. ABCA3 expression appeared to be low across a population of cells with high CSF-1R expression enriched from hemangiosarcoma cell lines when expression was examined using microarray anlaysis (J-H. Kim, unpublished observation). Thus ABCA3 may not contribute to lysosomal sequestration in hemangiosarcomas. In contrast, ABCB1 expression on lysosomes has been identified and may contribute to doxorubicin sequestration since doxorubicin accumulation was inhibited in the presence of the ABCB1 inhibitor valspodar [45]. Thus, further studies examining ABCB1 expression and localization in CSF-1R^low^ versus CSF-1R^high^ cells are warranted, and additional candidates may be identified through microarray analysis.

Although the hemangiosarcoma and angiosarcoma cell lines showed similar drug resistance mechanisms and these populations could be enriched by targeting CSF-1R expression, the cell surface marker expression profiles differed between species. Previous studies using hemangiosarcoma cell lines demonstrated expression of early hematopoietic (CD34, CD117, and CD113) and endothelial progenitor (CD34 and CD133) markers [6]. While expression of CD34 and CD117 by both the CSF-1R^low^ and CSF-1R^high^ cells was observed in the hemangiosarcoma cell lines, expression of CD133 was exclusive to the CSF-1R^high^ cell subpopulation. The retention of CD133 expression by the CSF-1R^high^ cell population brings up the intriguing possibility in hemangiosarcomas that the CSF-1R^high^ cell population possesses the potential for differentiation into CSF-1R^low^ cells and may be responsible for maintaining tumor growth autocrine or paracrine loops since we previously determined that the ligands for CSF-1R, CSF-1 and IL-34, are expressed by these and other canine hemangiosarcoma cell lines (B. H. Gorden, unpublished data). Studies using cell sorting of lineage markers or single cell isolation followed by clonal expansion would address both hypotheses, but our group has not yet undertaken these approaches.

In contrast, expression of CD34 and CD117 were observed mainly in the monolayer and the CSF-1R^low^ cell population from the AS5 cell line, although some expression was observed in the CSF-1R^high^ cells. We did not detect expression of CD133. Our results are similar to those reported by Lui *et al.* where the expression of both CD34 and CD117 was detected in angiosarcomas by immunohistochemistry. In keeping with our observations for CD133, detection of CD133 expression by immunohistochemistry was negative overall [9]. In both cases, the expression levels of CD133 may have been below the detection limit by immunohistochemistry due to cellular differentiation or tumor heterogeneity and this may also be the case for flow cytometry [9]. Regardless, the overall expression of these markers in the AS5 cell line reflects previous findings, and they also highlight that differences do exist between human angiosarcomas and canine hemangiosarcomas at the cellular and molecular level even though their overall pathologies appear to be virtually indistinct. Further studies are needed to characterize the potential differences as well as the noted similarities between these tumors.

## Conclusions

We present the finding that cells with high CSF-1R expression are more drug resistant than cells with low levels of CSF-1R expression in hemangiosarcoma and angiosarcoma cell lines. Investigation of the mechanistic basis of the selective resistance revealed increased lysosomal capacity in CSF-1R^high^ cells enriched from canine hemangiosarcoma and human angiosarcoma cell lines as a potential mechanism. Our results highlight drug resistance as a common feature in representative cell lines from both sarcomas, further justifying how studies in dogs may be especially relevant to understanding drug resistance mechanisms of the human disease. The identification of these populations also offers a unique model to study the development and progression of vascular sarcomas in addition to providing a way to predict responses to therapy and identify new treatment approaches.

## Abbreviations

AML: Acute myeloid leukemia; CSF-1: Colony stimulation factor 1; CSF-1R: Colony stimulating factor 1 receptor; DCV: DyeCycle Violet; TAM: Tumor associated macrophage.

## Competing interests

The authors declare that they have no competing interests.

## Authors’ contributions

BHG conceived parts of the project, contributed to the writing of the manuscript, performed the experiments using the canine hemangiosarcoma cell lines, and analyzed and prepared data for publication. JS performed the flow cytometry and cytotoxicity experiments for the AS5 cell line, generated the LysoTracker data, and helped analyze and prepare date for publication. AK assisted with some of the flow cytometry experiments, data analyses, and interpretation, GKS conceived parts of the project and edited the manuscript, and EBD conceived parts of the project, contributed to the writing of the manuscript, analyzed and interpreted data, and coordinated the project. All authors read and approved the final manuscript.

## Supplementary Material

Additional file 1: Table S1 Distribution of CSF-1R^high^ cells before and after the cell enrichment process. **Table S2.** Approximate EC_50_ and confidence interval values (95%) for the monolayer, CSF-1R^low^ and CSF-1R^high^ cell populations in response to doxorubicin treatment. **Table S3.** Intracellular LysoTracker fluorescence detected in CSF-1R cell populations presented as the range of fold change in CSF-1R^high^/CSF-1R^low^ relative fluorescent values (n = 2 for each cell line). Click here for file

Additional file 2: Figure S1 Assessment of CSF-1R expression after doxorubicin treatment. COSB cells were exposed to increasing concentrations of doxorubicin for 72 hours, and the remaining viable cells were assessed for the expression of CSF-1R by flow cytometry. 7-AAD was added to the cells before analysis to exclude nonviable cells. The overall percentages of CSF1-R^low^ and CSF-1R^high^ cells remaining after treatment is shown in **(A)** while the cell numbers remaining after treatment are shown in (B). **Figure S2.** Measurement of ABCB1 and ABCG2 expression by AS5 cells. Cell surface expression of ABCB1 and ABCG2 by AS5 cells was assessed using flow cytometry. Positive staining is indicated by the solid black lines, and the isotype controls are represented as shaded regions. MFI = mean fluorescence intensity. **Figure S3.** Measurement of CD34, CD117, and CD133 expression by AS5 cells. Cell surface expression of CD34, CD117, and CD133 was assessed using flow cytometry. Positive staining is indicated by the solid black lines, and the isotype controls are represented as shaded regions. MFI = mean fluorescence intensity. Click here for file
