# Supplementary material for: Lysosomal drug sequestration as a mechanism of drug resistance in vascular sarcoma cells marked by high CSF-1R expression
Source: Vasc Cell. 2014 Oct 1;6:20. doi: 10.1186/2045-824X-6-20 (PMC4188569; doi:10.1186/2045-824X-6-20)
Supplement: Additional file 1: Table S1 — Distribution of CSF-1Rhigh cells before and after the cell enrichment process. Table S2. Approximate EC50 and confidence interval values (95%) for the monolayer, CSF-1Rlow and CSF-1Rhigh cell populations in response to doxorubicin treatment. Table S3. Intracellular LysoTracker fluorescence detected in CSF-1R cell populations presented as the range of fold change in CSF-1Rhigh/CSF-1Rlow relative fluorescent values (n = 2 for each cell line). [file 2045-824X-6-20-S1.pdf]

Table S1. Distribution of CSF-1R^{high} cells before and after the cell enrichment process

Cell line	Subpopulation	% CSF-1R^{low}	% CSF-1R^{high}
**COSB**	Monolayer	98.4	1.68
	CSF-1R^{low}	96.6	3.41
	CSF-1R^{high}	77.4	22.5
**DD-1**	Monolayer	98.5	1.46
	CSF-1R^{low}	92.8	7.11
	CSF-1R^{high}	56.4	43.4
**AS5**	Monolayer	98.5	1.16
	CSF-1R^{low}	96.2	3.27
	CSF-1R^{high}	78.1	21.7

Table S2. Approximate EC_{50} and confidence interval values (95%) for the monolayer, CSF-1R^{low} and CSF-1R^{high} cell populations in response to doxorubicin treatment

Cell line	Cell population	Approximate EC_{50} [nM]	Confidence interval [nM]
**COSB**	Monolayer	104.6	60.1 – 181.9
	CSF-1R^{low}	65.1	40.3-105.4
	CSF-1R^{high}	197.4	136.1 - 285.8
**DD-1**	Monolayer	198.9	156.3 – 254.7
	CSF-1R^{low}	176.9	82.4 - 379.3
	CSF-1R^{high}	1255.1	638.3 - 2471
**AS5**	Monolayer	107.4	25.1 - 402.7
	CSF-1R^{low}	94.1	17.8 - 496.6
	CSF-1R^{high}	275.2	70.8 - 1069

Table S3. Intracellular LysoTracker fluorescence detected in CSF-1R cell populations presented as the range of fold change in CSF-1R^{high}/CSF-1R^{low} relative fluorescent values (n = 2 for each cell line)

Cell line	LysoTracker Concentration [nM]	Range of fold change (CSF-1R^{high}/CSF-1R^{low})	Mean
**COSB**	5	1.7-2.5	2.1
	10	1.4-1.5	1.5
	25	1.2-1.9	1.5
	50	1.4-1.6	1.5
	100	1.5-1.9	1.7
**AS-5**	5	1.3-1.7	1.5
	10	2.0-3.1	2.5
	25	1.6-1.9	1.8
	50	1.5-1.8	1.6
