# Supplementary figures and images for: Lysosomal drug sequestration as a mechanism of drug resistance in vascular sarcoma cells marked by high CSF-1R expression
Source: Vasc Cell. 2014 Oct 1;6:20. doi: 10.1186/2045-824X-6-20 (PMC4188569; doi:10.1186/2045-824X-6-20)

Figure S1

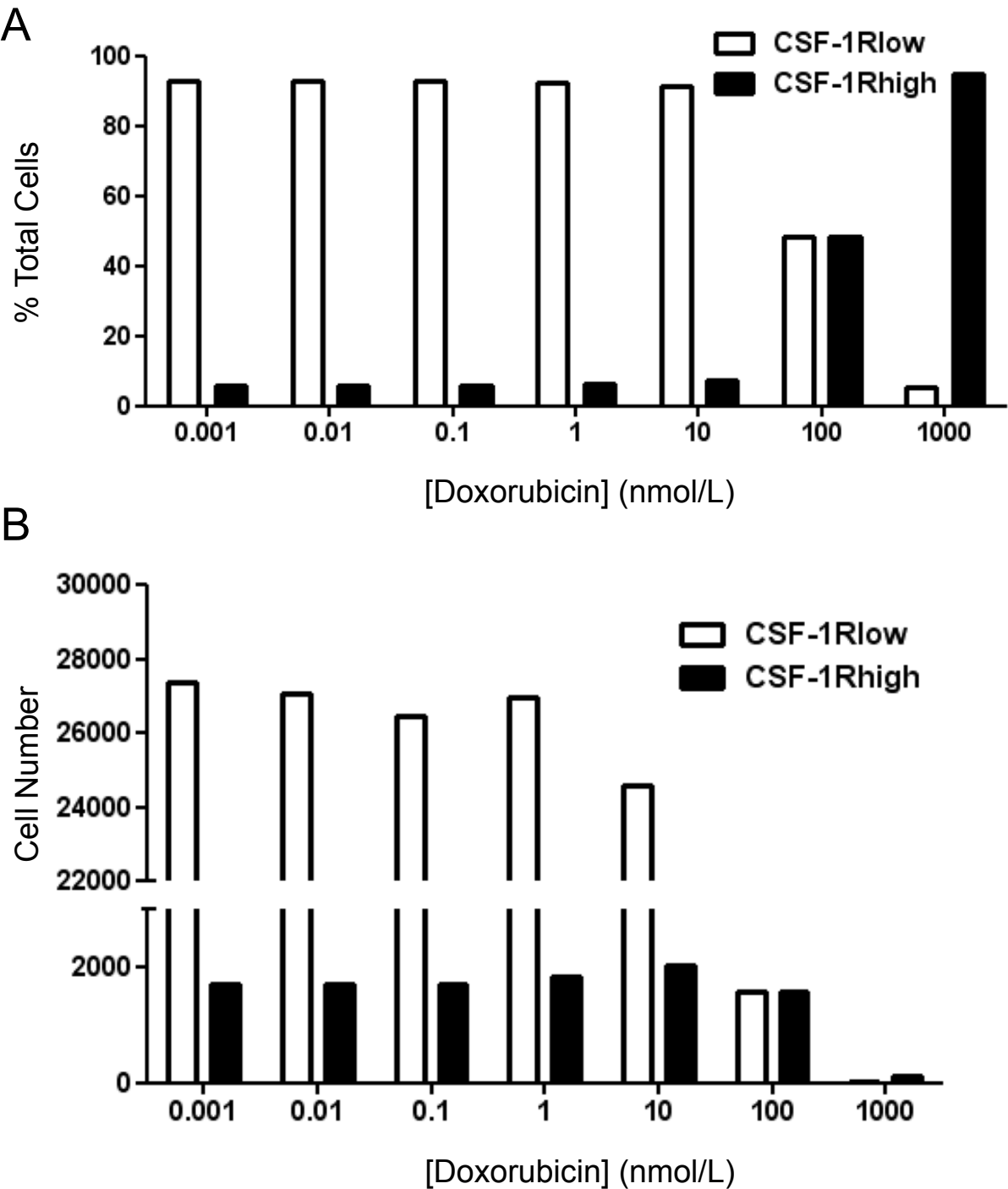

Figure S2

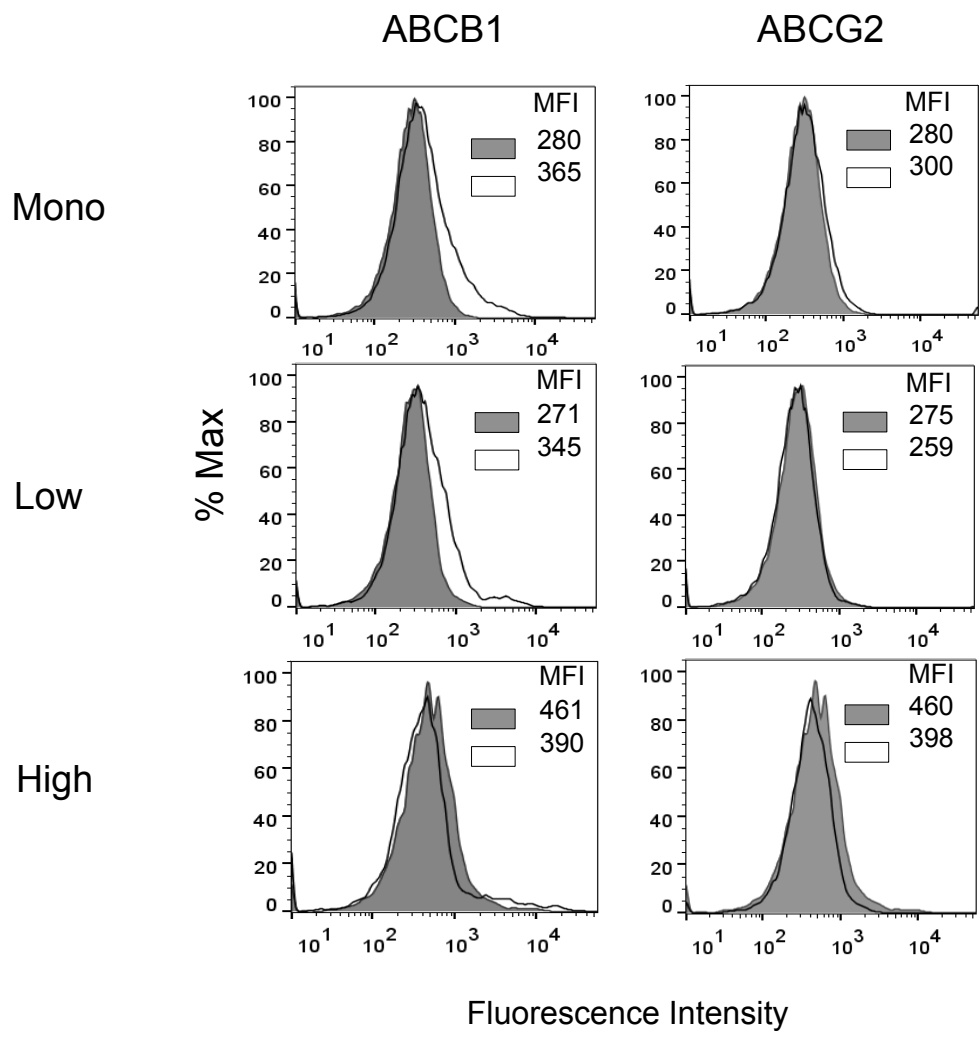

Figure S3

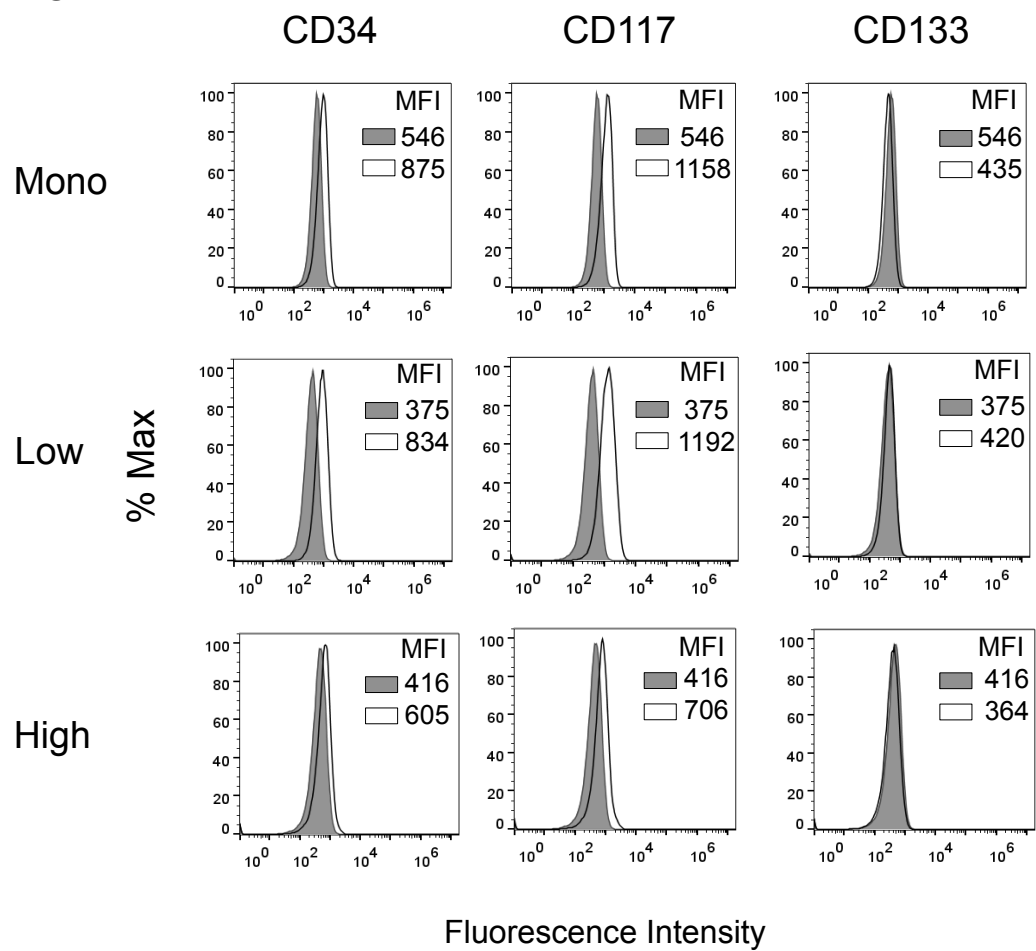

Supplement: Additional file 2: Figure S1 — Assessment of CSF-1R expression after doxorubicin treatment. COSB cells were exposed to increasing concentrations of doxorubicin for 72 hours, and the remaining viable cells were assessed for the expression of CSF-1R by flow cytometry. 7-AAD was added to the cells before analysis to exclude nonviable cells. The overall percentages of CSF1-Rlow and CSF-1Rhigh cells remaining after treatment is shown in (A) while the cell numbers remaining after treatment are shown in (B). Figure S2. Measurement of ABCB1 and ABCG2 expression by AS5 cells. Cell surface expression of ABCB1 and ABCG2 by AS5 cells was assessed using flow cytometry. Positive staining is indicated by the solid black lines, and the isotype controls are represented as shaded regions. MFI = mean fluorescence intensity. Figure S3. Measurement of CD34, CD117, and CD133 expression by AS5 cells. Cell surface expression of CD34, CD117, and CD133 was assessed using flow cytometry. Positive staining is indicated by the solid black lines, and the isotype controls are represented as shaded regions. MFI = mean fluorescence intensity. [file 2045-824X-6-20-S2.pdf]
